# Effects of Importin α1/KPNA1 deletion and adolescent social isolation stress on psychiatric disorder-associated behaviors in mice

**DOI:** 10.1371/journal.pone.0258364

**Published:** 2021-11-12

**Authors:** Koki Sakurai, Taichi Itou, Makiko Morita, Emiko Kasahara, Tetsuji Moriyama, Tom Macpherson, Takaaki Ozawa, Yoichi Miyamoto, Yoshihiro Yoneda, Atsuo Sekiyama, Masahiro Oka, Takatoshi Hikida

**Affiliations:** 1 Laboratory for Advanced Brain Functions, Institute for Protein Research, Osaka University, Osaka, Japan; 2 Department of Biological Sciences, Graduate School of Science, Osaka University, Osaka, Japan; 3 Department of Research and Drug Discovery, Medical Innovation Center, Kyoto University Graduate School of Medicine, Kyoto, Japan; 4 Advanced Pharmaco-science, Graduate School of Pharmaceutical Sciences, Osaka University, Osaka, Japan; 5 Faculty of Medical Sciences, Department of Cell Biology and Biochemistry, University of Fukui, Fukui, Japan; 6 Laboratory of Nuclear Transport Dynamics, National Institutes of Biomedical Innovation, Health and Nutrition, Osaka, Japan; 7 National Institutes of Biomedical Innovation, Health and Nutrition, Osaka, Japan; University of Queensland, AUSTRALIA

## Abstract

Importin α1/KPNA1 is a member of the Importin α family widely present in the mammalian brain and has been characterized as a regulator of neuronal differentiation, synaptic functionality, and anxiety-like behavior. In humans, a *de novo* mutation of the *KPNA1* (human Importin α5) gene has been linked with schizophrenia; however, the precise roles of KPNA1 in disorder-related behaviors are still unknown. Moreover, as recent studies have highlighted the importance of gene-environment interactions in the development of psychiatric disorders, we investigated the effects of *Kpna1* deletion and social isolation stress, a paradigm that models social stress factors found in human patients, on psychiatric disorder-related behaviors in mice. Through assessment in a behavioral battery, we found that *Kpna1* knockout resulted in the following behavioral phenotype: (1) decreased anxiety-like behavior in an elevated plus maze test, (2) short term memory deficits in novel object recognition test (3) impaired sensorimotor gating in a prepulse inhibition test. Importantly, exposure to social isolation stress resulted in additional behavioral abnormalities where isolated *Kpna1* knockout mice exhibited: (1) impaired aversive learning and/or memory in the inhibitory avoidance test, as well as (2) increased depression-like behavior in the forced swim test. Furthermore, we investigated whether mice showed alterations in plasma levels of stress-associated signal molecules (corticosterone, cytokines, hormones, receptors), and found that *Kpna1* knockout significantly altered levels of corticosterone and LIX (CXCL5). Moreover, significant decreases in the level of prolactin were found in all groups except for group-housed wild type mice. Our findings demonstrate that *Kpna1* deletion can trigger widespread behavioral abnormalities associated with psychiatric disorders, some of which were further exacerbated by exposure to adolescent social isolation. The use of *Kpna1* knockout mice as a model for psychiatric disorders may show promise for further investigation of gene-environment interactions involved in the pathogenesis of psychiatric disorders.

## Introduction

Importin αs, also known as Karyopherin αs (KPNAs), are a family of proteins that mediate nucleocytoplasmic transport in eukaryotic cells. They recognize and bind cytoplasmic cargo proteins containing nuclear localization signals (NLSs) and mediate their entry into the nucleus through formation of a Cargo-Importin α-Importin β1 trimeric complex [1]. Several members of the Importin α family are expressed widely across the brain [2], and have been linked with various human disorders of brain and behavior, including schizophrenia, mood disorders, and substance abuse [3–5]. Via individual subtypes that mediate the transport of different sets of cargo, importin αs can function as regulatory switches of gene expression [6], responsible for central cellular functions such as proliferation and differentiation [7–9]. Characterization of importin αs at a cellular level has revealed wide roles of importin α both inside and outside of canonical nucleocytoplasmic transport function [10–13]. However, further characterization is necessary to fully understand the implications of importin αs in physiological contexts.

In mice, importin α1 (human importin α5, gene symbol: *Kpna1*, protein symbol: KPNA1) is expressed widely throughout the central nervous system [2]. Past studies have demonstrated KPNA1 to be an important regulator of neuronal differentiation from mouse embryonic stem cells [7,8], although, interestingly, the brains of *Kpna1* knockout (KO) mice (labeled as importin α5-deficient mice by the authors, according to the human nomenclature) do not show any obvious morphological defects [14]. Behaviorally, *Kpna1* KO mice have been reported to exhibit reduced anxiety-like behaviors and impaired startle responses [15], indicating that KPNA1 may play a role in controlling behaviors associated with psychiatric disorders. Interestingly, in humans, examination of the exomes of schizophrenia patients have identified a *de novo* nonsense mutation in *KPNA1* (human importin α5 gene) [16], suggesting a possible link between KPNA1 and schizophrenia. These findings suggest the necessity for further characterization of the role of KPNA1 in controlling cognitive and behavioral functions known to be impaired in psychiatric disorders.

While gene sequencing studies in humans have identified several genetic risk factors for psychiatric disorders [17–19], increasing evidence suggests that interactions between genetic and environmental factors (G x E interaction) play important combinatorial roles in the development of psychiatric disorders [20–22]. Such findings support the “two-hit” hypothesis of schizophrenia, suggesting that the development of psychiatric disorders result from individuals with underlying genetic risk being exposed to environmental stress factors (e.g., social stress, malnutrition, or inflammatory episodes) [23]. Recent studies utilizing mouse models of psychiatric disorders have further supported this “two-hit” hypothesis by demonstrating that exposure to environmental stress factors can trigger or exacerbate behavioral deficits in genetically vulnerable mice [24,25]. These G x E interaction-associated behavioral alterations have been linked with various physiological alterations in the brain, including in intracellular signal transduction pathways [25], corticosteroid levels [24], and patterns of DNA methylation [24]. Such results demonstrate the effectiveness of G x E mouse models of psychiatric disorders as a tool for uncovering the roles of risk factors in psychiatric disorder pathogenesis, and suggest further use of such models will likely help to gain insight into the molecular pathology of psychiatric disorders.

In this study, we aimed to further characterize the physiological roles of KPNA1 in the brain by using an extensive behavioral test battery to analyze psychiatric disorder-related behaviors in a previously established *Kpna1* KO mouse line [26]. Moreover, we assessed the cumulative effects of an environmental risk factor in combination with *Kpna1* deletion by subjecting *Kpna1* KO mice to adolescent social isolation stress, a developmental stress paradigm proposed to model social stress factors found in human patients [24,27,28]. Finally, in order to identify potential biomarkers of psychiatric disorders, we assessed the effects of genetic and environmental factors on levels of signal molecules (corticosterone, cytokines, protein hormones) contained in plasma [29–31].

## Materials and methods

### Animals

#### Generation of *Kpna1* Het and KO mice

In this study, we used a previously reported *Kpna1* knockout line where Exons 2 and 3 have been removed [26] (deposited to RIKEN BioResource Research Center as RBRC06031; mice were referred to as Importin α5 KO (*Impα5*^-/-^) mice by the authors, according to the human nomenclature). The *Kpna1* knockout line was backcrossed >10 generations on a C57BL/6JJcl background prior to all experiments with mice purchased from CLEA Japan, Inc. (Tokyo, Japan). Homozygous *Kpna1* knockout (KO), and wild type (WT) mice were produced by mating male and female heterozygous *Kpna1* knockout mice.

#### Housing

Mice were kept in a noise-attenuating and temperature-controlled room at 23°C ± 2°C on a 12h light/dark cycle (Light: 0900–2100 Dark 2100–0900) with *ad libitum* access to standard mouse chow and fresh water.

Group-housed mice were kept in standard cages (21 x 32 x 13 cm) together with their same sex siblings (3–6 mice per cage) for the entire duration of experiments. Social isolation stress was performed as previously reported [24], with minor modifications. Isolated mice were separated from their siblings and housed individually in a small cage (length:20 x width:12.5 x height:11 cm) surrounded by white paper during the adolescent period (between ages 5 w to 8 w old). After isolation, mice were returned to standard cages with their same sex siblings.

All mouse experiment procedures were approved by the Institutional Safety Committee on Recombinant DNA Experiments (approval ID 04219), Animal Experimental Committee of Institute for Protein Research at Osaka University (approval ID 29-02-1), and the Animal Care and Use Committee of Kyoto University (approval ID MedKyo17071).

### Behavioral test battery

Behavioral tests were conducted after the mice reached 8 weeks old. All tests were conducted during the daytime (13:00–18:00) according to institutional regulations. The behavioral test battery consisted of the following tests administered in the following order: open field test (OFT), elevated plus maze (EPM), Y-Maze, novel object recognition test (NORT), inhibitory avoidance (IA), prepulse inhibition (PPI), and forced swim (FS). All tests were administered with 2 to 5 days in between tests.

A total of 39 male mice (group-housed: 9 WT, and 11 *Kpna1* KO; isolated: 10 WT, and 9 *Kpna1* KO) were subjected to the behavioral test battery. All behavioral tests were performed in the Medical Innovation Center, Kyoto University.

#### Open field test

Spontaneous locomotion was measured in the open field test. Mice were placed in the center of a grey plexiglass box (length:40 x width:40 x height:27 cm) located in a brightly illuminated sound-attenuating room and allowed to freely explore for 60 min. Novelty-induced locomotion was assessed by the total distance traveled (cm) and the percentage of time in the center area (1/3 of the width and length) of the field over the first 10 min of the 60 min session. Basal levels of locomotion beyond initial novelty-induced locomotion were assessed by measuring the total distance traveled (cm) in the box over 60 min. All measurements were scored using EthoVision XT 8.5 software (Noldus).

#### Elevated plus maze test

The elevated plus maze test was conducted as previously described [32], using a plus-shaped maze with 4 arms (each length:30 cm x width:7 cm), consisting of 2 open arms (arms without walls), 2 closed arms (arms with surrounding walls (height:20 cm), and a center area (length:7 cm x width:7 cm) connecting the arms. Mice were placed in the center area facing a closed arm and allowed to explore the maze for 15 min. The percentage of time spent in the open arms (%), and the number of entries into the open arms were scored using EthoVision XT 8.5 (Noldus). Mice were excluded from analysis if they fell from the open arms onto the floor below.

#### Y-maze test

The Y-maze test was conducted as previously described [33], using a Y-shaped maze with 3 arms (length:42 cm, wall height:15cm) spaced 120° apart from each other. Mice were placed in the center area and allowed to explore for 15min. Entries into the arms were scored using EthoVision XT 8.5 (Noldus). The alternation rate (%) was calculated using the following equation:

Alternationrate(%)=[no.oftimesall3armswereconsecutivelyentered]/([totalno.ofentries]-2)x100

#### Novel object recognition test

The novel object recognition test was conducted in an open field apparatus (length:40 x width:40 x height:27 cm). The mice were habituated to the field for 5 min for 3 consecutive days. On the 4th day, 2 identical objects (Object 1, Object 2) were placed inside the box and the mouse was allowed to explore for 5 min (training session) before being returned to their home cage. Fifteen min after the end of the training session the test session was begun. Mice were returned to the field where one of the objects was exchanged for a novel object (Object 1’) and allowed to explore for 5 min (retention session), during which time their movement was recorded on video. Mice were excluded from analysis if they spent more than 50% of the duration of a session on top of one of the objects. The amount of time the mice spent interacting with each object during the test session was scored using EthoVision XT 8.5 (Noldus), and the exploratory preference (%) for was calculated as follows:

ExploratoryPreference(%)=[InteractionwithObject1’]/([InteractionwithObject1’]+[InteractionwithObject2])*100

#### Inhibitory avoidance test

The step through inhibitory avoidance test was performed as previously described [34,35], using a two-chamber light-dark transition apparatus (Med Associates) consisting of an illuminated grey “light” chamber, a light-attenuating black “dark” chamber, and a sliding door between the two rooms. On day 1, mice were introduced into the “light” chamber and their latency (sec) to enter into the “dark” chamber (all four paws crossing the threshold of the door) was manually recorded using a stopwatch. Immediately after entry into the “dark” chamber, mice were subjected to a 1 sec footshock (0.5 mA) then left for 1 min in order for conditioning to occur, after which time they were returned to their home cages. On day 2, mice were introduced into the “light” chamber again, and their latency to enter into the “dark” chamber was measured. Mice were excluded from analysis if the latency to enter on day 1 exceeded 1 min.

#### Prepulse inhibition test and startle response

The prepulse inhibition test was administered as previously described [36,37]. Mice were placed in sound-attenuating startle response system chambers (SR-LAB©, San Diego Instruments) and habituated to a 70 dB white noise for 30 min. The test session began after the habituation step and consisted of 6 different trial types (5 types of prepulse-pulse trials, and 1 pulse-only trial). Six blocks of the 6 trial types were presented in a pseudorandomized order with each trial type only presented once each block. Seventy dB white noise was presented for the entire duration of the test. The total duration of each trial was 500 ms, starting with a 50 ms null period followed by a prepulse (20 ms; 74, 78, 82, 86, or 90 dB white noise). The startle stimulus (40 ms; 120 dB white noise) was presented after a 100 ms delay, and was followed by a 290 ms recording time. The pulse-only trial had no prepulse and only had the 70 dB background noise presented for the 20 ms period. The following formula was used to calculate the percentage (%) of prepulse inhibition (PPI):

(100-((startleresponsetoprepulse-pulse/startleresponseto120dBpulse)×100))

#### Forced swim test

The forced swim test was conducted as previously described [37] with minor modifications. Briefly, glass containers (diameter:13.5 cm x height:20 cm) were filled with room temperature (23-28°C) water up to a height of 14 cm. Mice were placed in the water for 6 min. The movement of the mice in the water was recorded on video and manually analyzed at a later date using a digital stopwatch to measure the time spent immobile (sec). Immobility was defined as a lack of any movement apart from those necessary to balance or keep the head above the surface.

### Blood plasma collection

Blood sampling was performed >5 days after the mice completed all steps of the behavioral test battery. Sampling was performed before noon (0900–1130) when basal corticosterone levels are low [38] to exclude the effects of circadian changes in corticosterone. Mice were deeply anesthetized with isoflurane then blood was removed from the inferior vena cava. Blood was immediately transferred to a 1.5 ml Protein LoBind tube (Eppendorf) containing 10 μl heparin sodium (10000 U/10mL Mochida Pharmaceutical, Tokyo) to prevent clotting. The blood was centrifuged at 1000 g for 10 min at 4°C to separate and collect plasma.

### Plasma Corticosterone measurement

Plasma Corticosterone levels were measured using a Corticosterone ELISA kit (Cayman Chemical Company) according to the manufacturer’s instructions. All measurements were in duplicate. Plasma samples from a total of 37 mice (group-housed: 9 WT, 9 *Kpna1* KO; isolated: 10 WT, and 9 *Kpna1* KO) with enough volume for measurement were used for ELISA measurement.

### Plasma cytokine measurement

Plasma levels of cytokines were measured using a Mouse Magnetic Luminex Assay (R&D systems), according to the manufacturer’s instructions. Twenty-nine different cytokines were measured: IL-1 β, IL-2, IL-4, IL-5, IL-6, IL-7, IL-10, IL-13, IL-16, IL-17A, IL-17E, IL-33, IL-6 Rα, CCL3, CCL5, CCL11, CXCL 1, CXCL10, LIX, IFN-g, TNF-α, TNF RI, TNF RII, VEGF, PDGF-BB, Prolactin, TIMP-1, G-CSF, and GM-CSF. Plasma samples from a total of 35 mice (group-housed: 9 WT, 9 *Kpna1* KO; isolated: 9 WT, and 8 *Kpna1* KO) with enough volume for measurement were measured in the multiplex immunoassay.

### Statistical analysis

All statistical analyses and data visualization were performed using Prism 8.0 (GraphPad Software, La Jolla, CA). Data are presented as the Mean ± SEM for bar graphs, with dots indicating individual data points. For box-whisker plots, data is presented as the median (center line), ±1.5 interquartile range (box), and minimum and maximum values (whiskers). For all experiments other than the NORT, IA, and PPI, the main effects of genotype and environment, and their interaction, were analyzed with a 2-way analysis of variance (ANOVA). The main effects of genotype, environment, and trial, and their interactions, in the IA and PPI tests were analyzed with a 3-way repeated measures ANOVA. For PPI, Greenhouse-Geisser-corrected degrees of freedom were used for the main effect of the trial. All ANOVA results are shown in S1 Table. When a significant interaction between genotype and environment was observed in the ANOVA analysis, a post hoc Tukey’s multiple comparisons test was used to analyze differences between groups differing by 1 factor. Differences between the two groups in the NORT were analyzed with an unpaired t test. Outliers were determined by the ROUT test (Q:1%) on Prism 8.0. The number of individual mice used for analysis, as well as the number of excluded outliers are shown in S2 Table.

## Results

### Behavioral test battery

To investigate the effects of *Kpna1* deletion and adolescent isolation on psychiatric-disorder related behaviors, we designed a behavioral test battery to assess anxiety-like behaviors(OFT, EPM), Memory (Y-Maze, NORT, IA), sensorimotor gating (PPI), and depression-like behavior (FS). Two similar-sized groups were either group-housed or subjected to social isolation during adolescence (isolated). A total of 9 WT and 11 *Kpna1* KO group-housed mice, and 10 WT and 9 *Kpna1* KO socially isolated mice, were subjected to the behavioral test battery.

### Anxiety-like behaviors

#### Open field test

**Fig 1:**
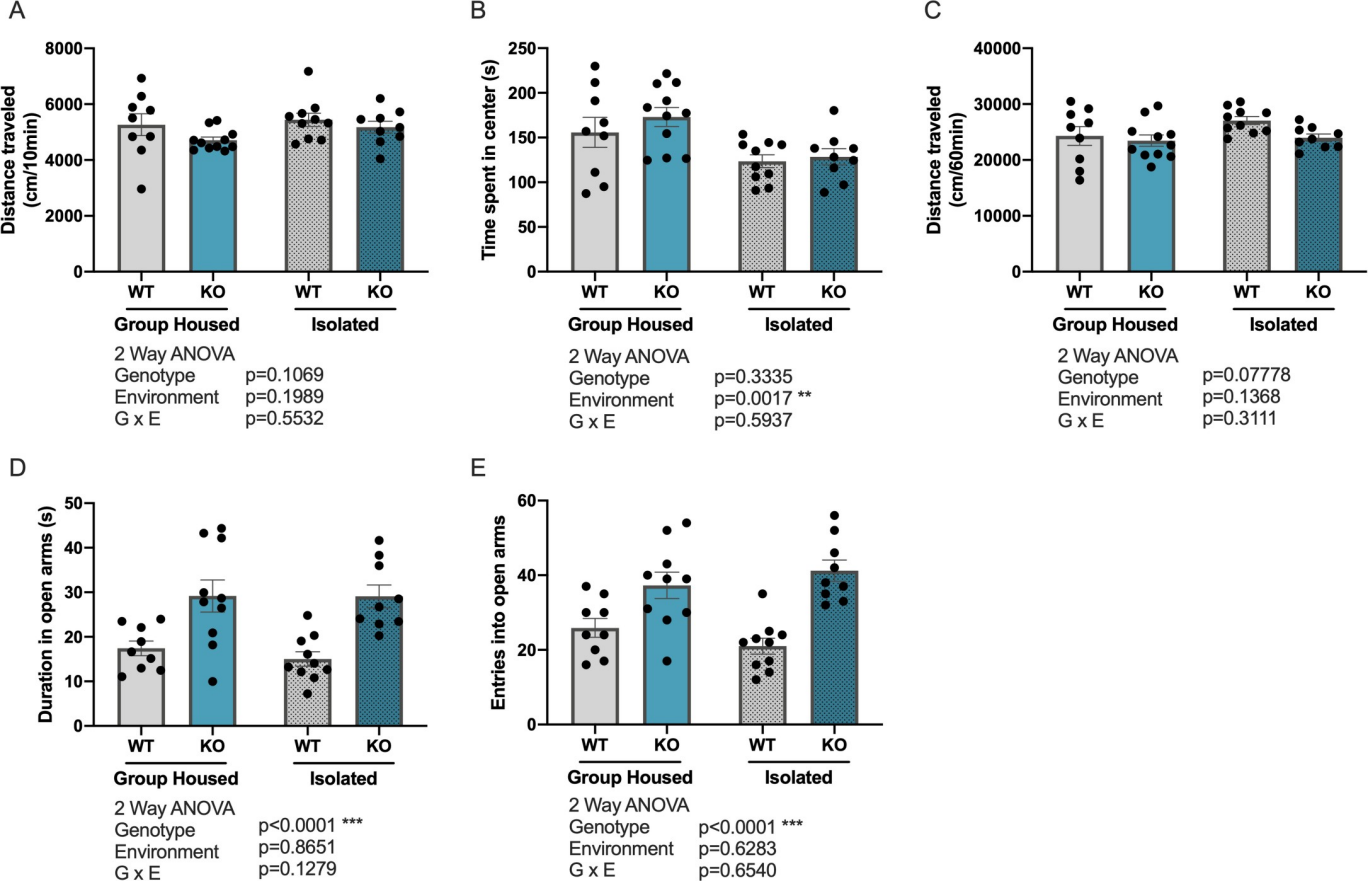
*Kpna1* KO mice show decreased anxiety-like behavior in the elevated plus maze. Assessment of Anxiety-like behaviors in group-housed and isolated WT and *Kpna1* KO mice. (A-C) Open field test (A) Total distance traveled in the open field over 10 min (cm). (B) Time (s) spent in the center of the open field over 10 min. (C) Total distance traveled in the open field over 60 min (cm). (D-E) Elevated plus maze (EPM) (D) Total number of entries into the open arms in the EPM test. (E) Time (s) spent in the open arms in the EPM test. Mean ± SEM. Group-housed: 9 WT, 11 *Kpna1* KO; isolated: 10 WT, 9 *Kpna1* KO.

The open field test is a behavioral test used to assess spontaneous locomotion and anxiety-like behavior in a novel environment [39]. The first 10 min of the 60 min trial was used to assess novelty induced locomotion by calculating the total distance traveled over 10 min (Fig 1A). Anxiety-like behavior was assessed by measuring the time spent in center of the field (Fig 1B) during the first 10 min. Two-way ANOVA analysis showed no significant main effect of either genotype or environment on the distance traveled over 10 min. A significant main effect of environment (F _(1,35)_ = 11.51, p = 0.0017) was seen in in the duration of time spent in the center of the field, with isolated mice spending less in time in the center compared to group-housed mice, indicating higher levels of anxiety-like behavior in isolated mice. The total distance traveled over the entire 60 min duration of the test was used to assess basal levels of locomotion beyond the initial novelty induced locomotion (Fig 1C). Two-way ANOVA analysis on the total distance traveled over 60 min showed no significant main effect of genotype or environment.

#### Elevated plus maze test

Next, we examined behavior in an elevated plus maze (EPM), a test for assessing anxiety-like behavior in rodents [40]. The total number of entries into, as well as the total time spent in the open arms was measured, where more exploration of the open arms indicates a decrease in anxiety-like behavior. Two-way ANOVA analysis showed a significant main effect of genotype on both the duration of time (Fig 1D; F_(1,34)_ = 25.92, p<0.001) and number of entries (Fig 1E; F_(1,34)_ = 31.40, p<0.001) spent in the open arms, with *Kpna1* KO showing an increase in both measures compared with WT controls. There was no significant main effect of environment on both the total entries into open arms and total time spent in open arms.

### Memory-related tasks

#### Y-maze test

**Fig 2:**
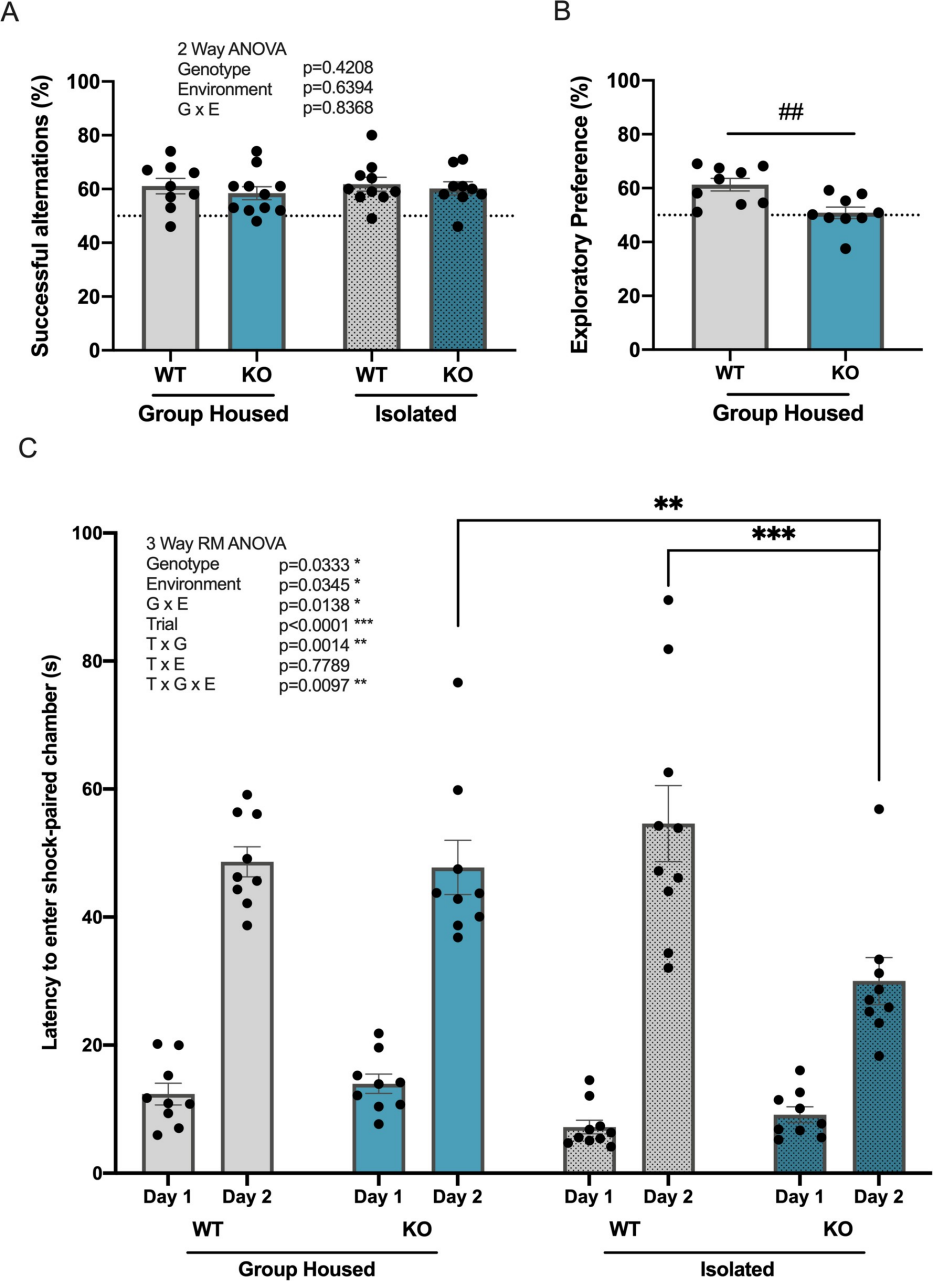
*Kpna1* KO mice show significant impairment in short-term recognition memory and avoidance learning and/or memory. Assessment of spatial working memory (Y-maze), object recognition memory (novel object recognition test (NORT)) and aversive learning and/or memory (inhibitory avoidance (IA)) in group-housed and isolated WT and *Kpna1* KO mice. (A) Y-maze (A) Rate of correct alternations (%) in the Y-maze. (B) NORT (B) Exploratory preference of the novel object (%) in the NORT during the test session. (C) IA (C) Latency (s) to enter into the shock-paired chamber on Day 1 (pre-learning) and Day 2 (post-learning) in IA. Dotted line indicates 50% chance value. Mean ± SEM, ## p < 0.01 unpaired t test; ** p < 0.01, *** p < 0.001 Tukey’s test. (A-B) Group-housed: 9 WT, 11 *Kpna1* KO; isolated: 10 WT, 9 *Kpna1* KO (C) Group-housed: 9 WT, 9 *Kpna1* KO (D) Group-housed: 9 WT, 9 *Kpna1* KO; isolated: 10 WT, 9 *Kpna1* KO.

Short-term spatial memory was assessed using a Y-maze test of spontaneous alternation [41]. In the Y-maze test, a decrease in the percentage of successful alternations between arms indicates impaired spatial working memory. All 4 groups of mice showed higher levels of successful alternations than the 50% rate expected by chance (Fig 2A). Two-way ANOVA analysis showed no significant main effect of genotype or environment on the number of correct alternations between groups.

#### Novel object recognition test

The ability for short-term recognition memory was evaluated using the novel object recognition test (Fig 2B) [42]. An exploratory preference for the novel object during the test session 15 min after the training session suggests successful recognition of the familiar object. During both sessions, all isolated mice (isolated WT and *Kpna1* KO) climbed on top of the objects and spent extended periods of time (>50% session duration) on them, resulting in them being excluded from the analysis. Analysis of group-housed mice revealed that *Kpna1* KO mice showed a significantly lower exploratory preference for the novel object in the test session (unpaired t test, t_(16)_ = 3.322, p = 0.0043).

#### Inhibitory avoidance test

The inhibitory avoidance test assesses aversive learning and memory by measuring the latency to enter into a ‘dark’ chamber paired with an aversive footshock stimulus (Fig 2C) [32,43]. Three-way repeated measures ANOVA analysis revealed a significant main effect of trial (day) on the latency to enter the ‘dark’ chamber (F _(1,34)_ = 265.1, p<0.001), indicating that mice were able to learn the contingency between the chamber and the shock with which it had been paired on day 1. Additionally, there were found to be significant main effects of genotype (G) (F _(1,34)_ = 4.920, p = 0.0333) and environment (E) (F _(1,34)_ = 4.852 p = 0.0345), as well as significant interactions between G x E (F _(1,34)_ = 6.741, p = 0.0138), Trial x G (F _(1,34)_ = 12.05, p = 0.0014), and Trial x G x E (F _(1,34)_ = 7.521, p = 0.0097). A post hoc Tukey’s test was used to analyze differences amongst each group and day. All 4 groups showed significant increases in latency to enter on day 2 compared to day 1 (group-housed: WT p<0.001, *Kpna1* KO p<0.001; isolated: WT p<0.001, *Kpna1* KO, p<0.001). Isolated *Kpna1* KO mice showed a significant decrease in latency to enter on day 2 compared to group-housed *Kpna1* KO and isolated WT mice (p = 0.0027; KO vs isolated KO, p = 0.0061; isolated WT vs isolated KO, p<0.001)

### Prepulse inhibition and startle response

**Fig 3:**
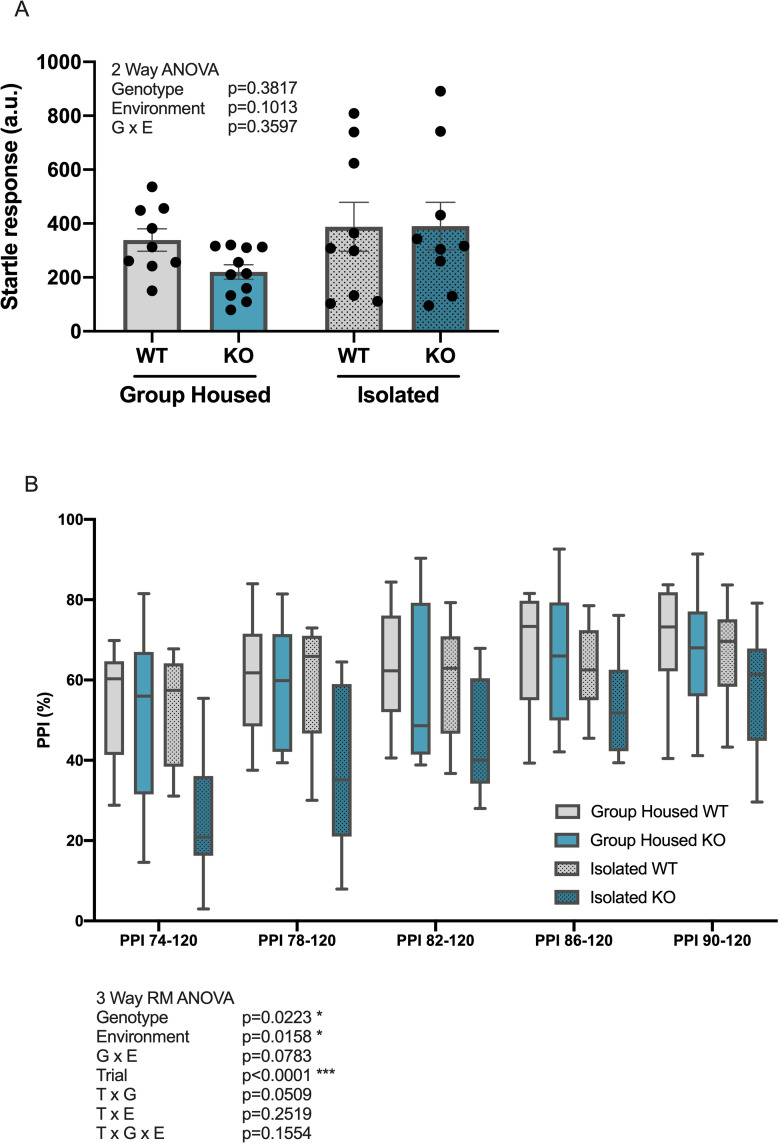
*Kpna1* KO mice show impaired sensorimotor gating in the prepluse inhibition test. Assessment of startle response and prepulse inhibition (PPI) in group-housed and isolated WT and *Kpna1* KO mice. (A) Startle response (a.u.) to a 120 dB startle pulse. (B) PPI (%) to 5 different prepulse strength levels (74, 78, 82, 86, 90 dB). (A) Mean ± SEM (B) median (center line), ±1.5 interquartile range (box), minimum and maximum values (whiskers). Group-housed: 9 WT, 11 *Kpna1* KO; isolated: 9 WT, 9 *Kpna1* KO.

The ability for sensorimotor gating was assessed in a PPI test, a cross-species measure known to be disrupted by schizophrenia and other disorders of the brain [44–48]. There was no significant main effect of genotype or environment on the startle responses to a 120 dB startle pulse (Fig 3A). Three-way repeated measures ANOVA analysis revealed a significant effect of trial (prepulse dB level) on the PPI percentage (F_(3.319,109.5)_ = 29.39, p<0.001, Geisser-Greenhouse’s epsilon = 0.8297), with PPI percentage increasing with louder prepulse (Fig 3B). Moreover, significant effects of genotype (F_(1,33)_ = 5.747, p = 0.0223) and environment (F_(1,33)_ = 6.473, p = 0.0158) on the PPI percentage were also observed, with *Kpna1* KO mice and isolated mice showing a decreased PPI percentage compared with their WT and group-housed counterparts, respectively.

### Forced swim test

**Fig 4:**
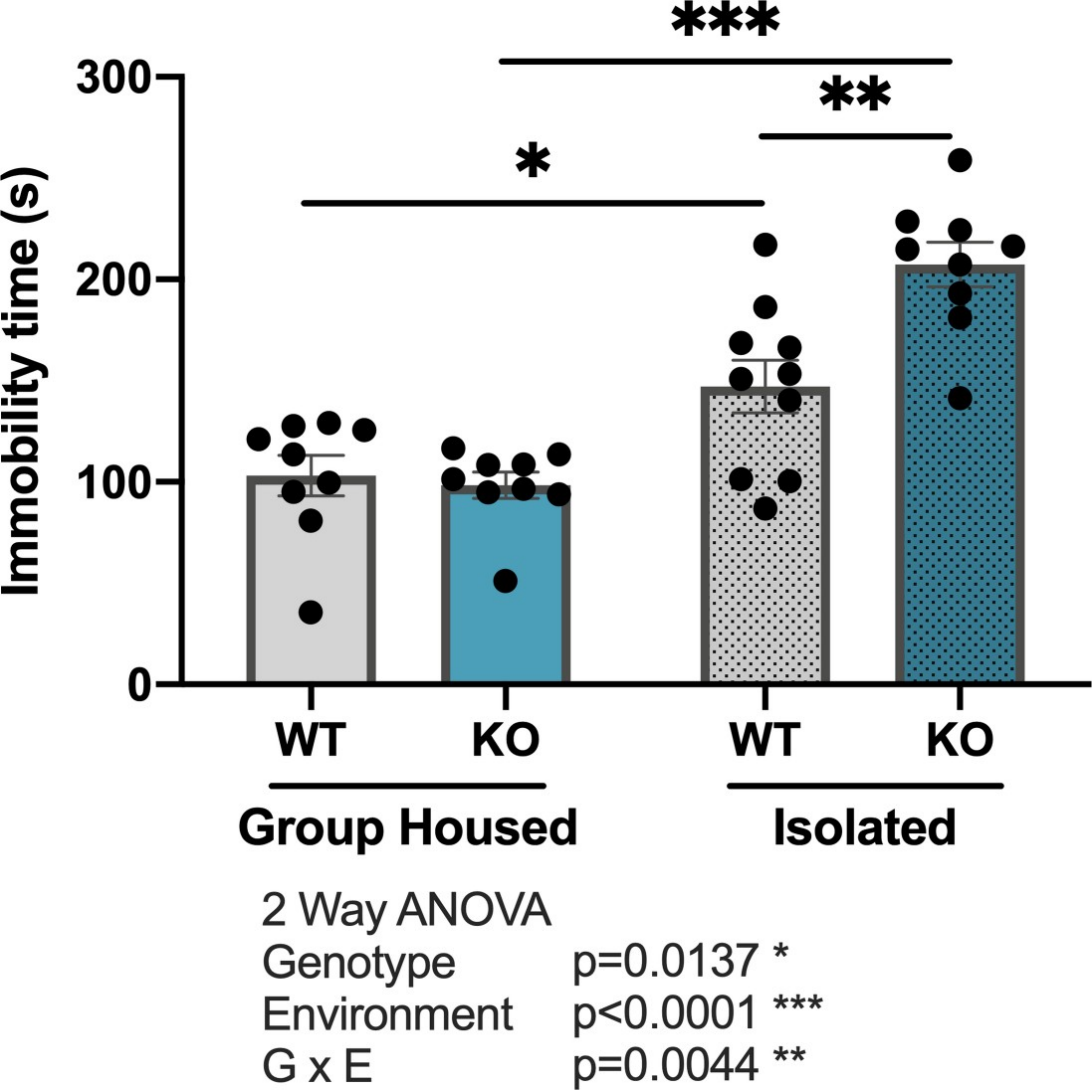
*Kpna1* KO mice show increased depression-like behavior in the forced swim test. Assessment of depression-like behaviors in the forced swim test (FS) in group-housed and isolated WT and *Kpna1* KO mice. Time spent immobile (s) in the FS. Mean ± SEM, * p < 0.05, ** p < 0.01, *** p<0.001 Tukey’s Test. Group-housed: 9 WT, 9 *Kpna1* KO; isolated: 10 WT, 9 *Kpna1* KO.

Immobility time in the FS test is often used to assess depression-like behavior in rodents [49]. Two-way ANOVA analysis revealed significant main effects of genotype (Fig 4; F _(1,33)_ = 6.789, p = 0.0137) and environment (F _(1,33)_ = 51.78, p<0.001), as well as a significant G x E interaction (F _(1,33)_ = 9.323, p = 0.0044), on the time spent immobile during the FS test (Fig 4). Post-hoc Tukey’s tests revealed that increased immobility time in isolated mice (group-housed WT vs isolated WT (p = 0.0271), group-housed *Kpna1* KO vs isolated KO (p<0.001)) was augmented by *Kpna1* KO (isolated WT vs isolated KO (p = 0.0016)).

In summary, both *Kpna1* deletion and adolescent social isolation stress were found to significantly impair aversive learning and/or memory in the IA test and sensorimotor gating in the PPI test, while increasing anxiety-like behavior (in the EPM for *Kpna1* deletion and OFT for isolation) and depression-like behavior in the FS test. Conversely, only *Kpna1* deletion was found to disrupt short-term recognition memory. Finally, gene x environment interaction in isolated *Kpna1* KO mice was observed in aversive learning and/or memory in the IA test as well as depression-like behavior in the FS test, where significant differences between WT and KO were only observed when the mice were subjected to social isolation stress.

### Assessment of plasma levels of stress-associated molecules

Characterization of group-housed and isolated *Kpna1* KO mice using a behavioral test battery showed that *Kpna1* deletion and social isolation stress both significantly alter several different psychiatric disorder-related behaviors. Moreover, a G x E interaction between *Kpna1* deletion and social isolation stress was observed in aversive learning and/or memory impairments and depression-like behavior in the IA and FS tests, respectively. As the administration of both genetic and environmental risk factors have previously been reported to cause major increases the levels of plasma corticosterone compared to when each risk factor is administered alone [24], we assessed plasma corticosterone levels of both group-housed and isolated groups after behavioral experiments. Additionally, as changes in neuroendocrine signaling through glucocorticoids is known to alter downstream levels of signal molecules in circulation following exposure to stress [29], we assessed the plasma levels of cytokines, hormones, and receptors in group-housed and isolated *Kpna1* KO and WT mice using a multiplex immunoassay after the completion of behavioral experiments [31].

#### Corticosterone ELISA

**Fig 5:**
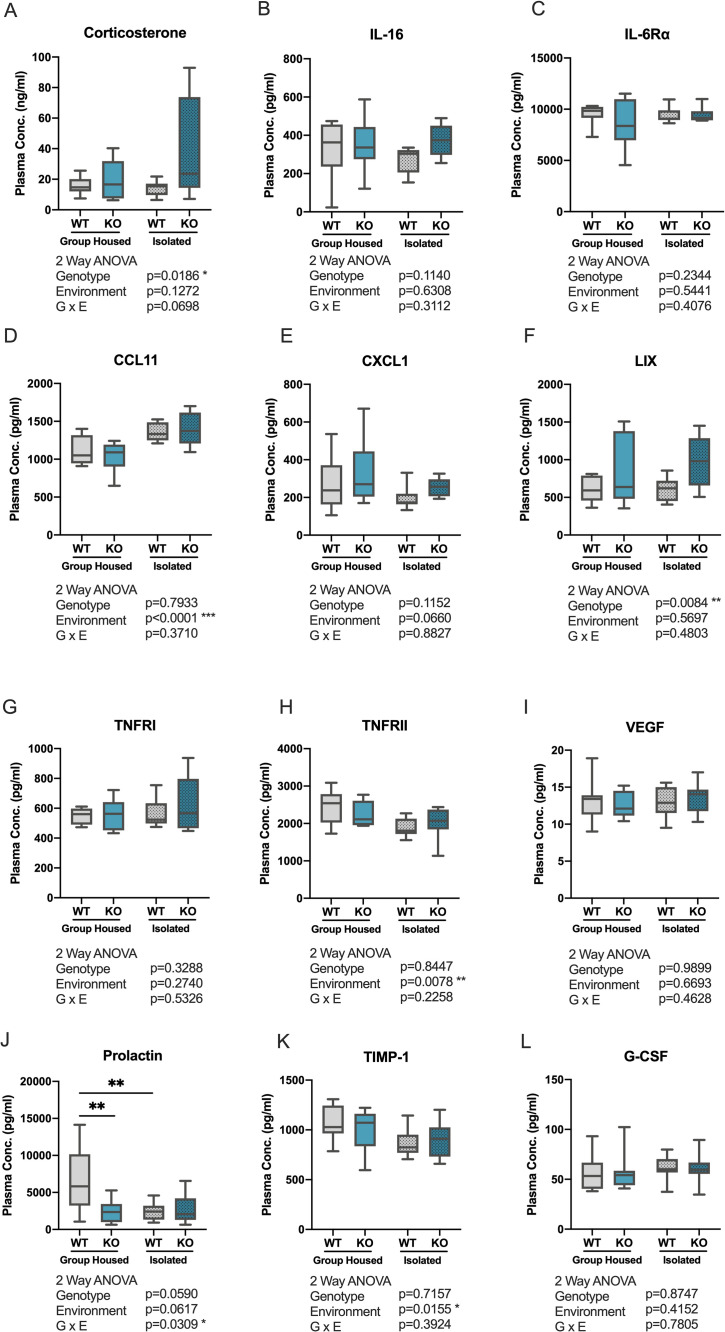
Measurement of stress-associated signal molecules in plasma. Measurement of plasma levels of stress-associated molecules by (A) ELISA or (B-L) multiplex immunoassay in group-housed and isolated WT and *Kpna1* KO mice. (A) Corticosterone, (B) IL-16, (C) IL-6Rα, (D) CCL11, (E) CXCL1, (F) LIX, (G) TNFRI, (H) TNFRII, (I) VEGF, (J) p, (K) TIMP-1, (L) G-CSF. ** p < 0.01 Tukey’s Test. Median (center line), ±1.5 interquartile range (box), minimum and maximum values (whiskers).

We used an ELISA assay to assess the plasma levels of corticosterone, a stress-related glucocorticoid [24,50–52] across group-housed and isolated *Kpna1* KO and WT mice (Fig 5A). After exclusion of outliers, the effects of genotype (*Kpna1* KO or WT) and/or environment (adolescent isolation or group-housing) on plasma corticosterone levels were analyzed using a 2-way ANOVA. A significant main effect of genotype was observed on plasma corticosterone levels (F_(1,32)_ = 6.148, p = 0.0186), with *Kpna1* KO mice demonstrating an increase in corticosterone levels compared with WT controls.

#### Multiplex immunoassay for cytokines, hormones, and receptors

Alterations in plasma glucocorticoid levels are known to affect neural activity, causing alterations in the activity of the Hypothalamus-Pituitary-Adrenal (HPA) signaling pathway. Moreover, such alterations can affect downstream pathways causing changes in systemic levels of signal molecules such as cytokines [53]. To assess the possible effects of *Kpna1* deletion and adolescent social stress on systemic levels of cytokines, hormones, and receptors, we used a multiplex bead-based immunoassay to measure the levels of cytokines (cytokines, hormones, and receptors) in plasma collected from mice subjected to the behavioral battery. Of the 29 species measured in the assay (IL-1 β, IL-2, IL-4, IL-5, IL-6, IL-7, IL-10, IL-13, IL-16, IL-17A, IL-17E, IL-33, IL-6 Rα, CCL3, CCL5, CCL11, CXCL 1, CXCL10, LIX, IFN-g, TNF-α, TNF RI, TNF RII, VEGF, PDGF-BB, Prolactin, TIMP-1, G-CSF, GM-CSF), 11 species (IL-16, IL-6 Rα, CCL11, CXCL 1, LIX, TNFRI, TNFRII, VEGF, Prolactin, TIMP-1, G-CSF) were detected above the lower limit of quantitation (LLQ) in plasma collected from both group-housed and isolated groups.

After exclusion of outliers, the effects of genotype (*Kpna1* KO or WT) and environment (adolescent isolation or group-housing), as well as their interactions, on plasma levels were analyzed using a 2-way ANOVA. A significant main effect of genotype was observed on LIX levels (Fig 5F, F_(1,31)_ = 7.931, p = 0.0084), with *Kpna1* KO mice showing increased levels compared with WT controls. Additionally, a significant main effect of environment was observed on levels of CCL11 (Fig 5D; F_(1,31)_ = 22.48, p<0.001), TNFRII (Fig 5H; F_(1,28)_ = 8.209, p = 0.0078), and TIMP-1 (Fig 5K; F_(1,31)_ = 6.567, p = 0.0155), with isolated mice demonstrating higher levels of CCL11, but lower levels of TNFRII and TIMP-1 than WT controls. Finally, a significant G x E interaction was observed for prolactin levels (Fig 5J; F_(1,28)_ = 5.168, p = 0.0309). Post hoc Tukey’s tests on the differences in prolactin amongst groups showed that WT mice had significantly higher levels of prolactin compared to *Kpna1* KO and isolated WT mice (WT vs KO, p = 0.0216; WT vs isolated WT, p = 0.0224). No significant effects of environment or genotype were observed on levels of IL-16 (Fig 5B), IL-6Rα (Fig 5C), CXCL1 (Fig 5E), TNFRI (Fig 5H), VEGF (Fig 5I), and G-CSF (Fig 5L).

In summary, *Kpna1* deletion was found to increase levels of corticosterone and LIX, regardless of whether animals were exposed to social isolation. However, genetic and environmental factor were found to interact in the control of prolactin levels, where group-housing was able to confer protection to WT mice against the reduced prolactin levels observed in group-housed *Kpna1* KO mice and isolated WT mice. Finally, isolation stress was found to increase plasma levels of CCL11 and decrease TNFRII and TIMP-1 in both WT and *Kpna1* KO mice.

## Discussion

In this study, we investigated the effects of *Kpna1* deletion and social isolation stress on psychiatric disorder-related behaviors by exposing group-housed and isolated *Kpna1* KO mice to an extensive behavioral test battery. We also assessed plasma levels of multiple stress-associated signal molecules in group-housed and isolated *Kpna1* KO mice. In our study, we found Kpna1 deletion decreases anxiety-like behavior, impairs short-term memory and sensorimotor gating, and increases depression-like behavior, suggesting that hypofunction of KPNA1 results in behavioral changes associated with psychiatric disorders [32,39–45,49]. Furthermore, the administration of adolescent social isolation stress, an environmental risk factor known to interact with genetic risk factors in the development of psychiatric disorders [25,27], resulted in significant impairment of aversive learning and/or memory in the IA and increased depression-like behavior in the FS in *Kpna1* KO mice. All behavioral alterations caused by *Kpna1* deletion and G x E interaction, other than decreased anxiety-like behavior, were identified here for the first time.

In the EPM, *Kpna1* KO mice demonstrated a significant decrease in anxiety-like behavior compared with WT mice. A previous study has reported that *Kpna1* KO mice show decreased anxiety-like behavior (in EPM and OFT), which was attenuated by either administration of a Sphk1 inhibitor PF-543, or rescue of *Kpna1* in the ventral hippocampus [15]. Interestingly, while there was a significant effect of social isolation observed in the time spent in the center of the field in the OFT in our study, no significant effect of *Kpna1* deletion was observed. The apparent contradiction between the OFT results in our study and those reported in the previous study may reflect differences between background strains of the *Kpna1* KO mice used (C57BL/6JJcl vs C57BL/6OlaHsd). Indeed, significant differences in the behavioral characteristics of different C57BL/6 substrains have been previously described [54,55]. Additionally, differences in length of social isolation stress [27], along with differences in experimental apparatus and procedures (ambient illumination, wall color) [56,57] are known to influence performance in both OFT and EPM, and the differences in results could reflect such differences in apparatus and procedure. Further investigation into molecular alterations following social isolation stress may provide insight on the molecular mechanisms regulating anxiety-like behaviors.

Here we show for the first time that *Kpna1* deletion causes significant impairment of PPI. PPI is frequently used as a cross-species measure of sensorimotor gating in a wide range of organisms [46–48], and is of particular interest for the development of rodent models of schizophrenia as PPI impairment is seen in both human schizophrenia patients [44] and various mouse models of schizophrenia [45]. Although the neural circuits that control sensorimotor gating are not yet fully understood, cortical and limbic pathways of the brain have been implicated in its control, suggesting that PPI impairment in *Kpna1* KO mice may reflect disfunction of such pathways [47,58]. A previous study has also reported that *Kpna1* deletion results in a significant decrease in acoustic startle response [15]. However, in our behavioral test battery, we did not observe any changes in acoustic startle to a 120 dB startle pulse in *Kpna1* KO mice. This apparent contradiction may also be the explained by differences in background strains or experimental protocols; our assessment of acoustic startle response was performed as a part of the prepulse inhibition test rather than on its own, as in the previous study [15].

In the FS, an increased depression-like phenotype was seen only in isolated *Kpna1* KO mice. This is the first report of increased depression-like behavior in *Kpna1* deficient mice, and stands in contrast to the decreased anxiety-like behavior observed in the EPM. Interestingly, there have been several studies reporting negative correlations between measures of anxiety-like behavior (OFT, EPM) and depression-like behavior (FS) [59,60]. This has inspired recent discussion about negative correlations between anxiety-like and depression-like behavior in mouse models of psychiatric disorders, and highlights the need for greater elucidation of the mechanistic background of anxiety-like and depression-like behaviors [61].

In the IA task, only isolated *Kpna1* KO mice exhibited reduced latency to enter into a shock-paired chamber compared to group-housed KO and isolated WT mice, indicating impaired aversive learning and/or memory. Other than impairments in aversive learning and/or memory, a decreased latency in the IA could suggest that isolated *Kpna1* KO mice have a reduced pain response compared to other groups. A recent study assessing the effect of different importin α deficiencies on pain responses in mice showed that *Kpna1* deletion (referred to as importin α5 in the manuscript) does not alter pain perception [62]. Therefore, it is unlikely that the decreased latency to enter the shock-paired chamber observed in *Kpna1* KO mice in this study reflects a reduced pain response, but rather impaired aversive learning and/or memory. In the NORT, a short-term memory-related task, group-housed *Kpna1* KO mice showed significantly fewer interactions with the novel object compared to WT controls. This is the first report of *Kpna1* deletion resulting in impaired novel object recognition. *Kpna1* deletion has been suggested to alter gene expression and decrease synaptic functionality of the hippocampus [15]. The hippocampus plays a central role in multiple types of memory including aversive memory [43], and in object recognition memory [63,64] together with the perirhinal cortex [65]. It is possible that the impairment in IA and NORT reported in our study may reflect memory disfunctions of the hippocampus caused by *Kpna1* deletion. However, it is important to note that in the IA test it is difficult to identify whether the reduced latency to enter the shock-paired chamber is the result of impaired aversive learning or impaired aversive memory, as the effects of genetic and environmental factors were present during both the acquisition (Day 1) and expression (Day 2) stages of the test. Indeed, while the hippocampus has been implicated in aversive memory [43], the ventral striatum appears to be important for aversive learning [34].

In the FS and IA tests, G x E interaction between *Kpna1* deletion and social isolation was found, where significant changes in behavior were only observed when adolescent social isolation and *Kpna1* deletion were both present. Such G x E interactions seen in these behaviors may suggest that environmental factors (adolescent social isolation) influence similar regions/pathways of the brain as genetic risk factors (*Kpna1* deletion), where the cumulative effects from both factors cause increased levels of impairment. Administration of social isolation stress has been shown to alter performance in FS along with increases in serum corticosterone levels and hippocampal microglial activation [66]. Similar increases in serum corticosterone levels have been found in mice with decreased IA performance after social isolation [67]. As increased plasma corticosterone is known to alter synaptic states in the hippocampus [68], increased glucocorticoid signaling acting onto the already altered *Kpna1* deficient hippocampus may be one possible mechanism behind the G x E interactions in behavior observed in this study. Further determinations of the individual regions/pathways susceptible to each genetic and/or environmental stress factor will allow for detailed examinations of the cellular processes in each region that result in the disfunctions of brain and behavior [50,51].

In our assessment of plasma corticosterone, *Kpna1* KO mice had higher corticosterone levels compared to WT mice. Corticosterone is a stress-associated steroid hormone analogous to human cortisol, which contributes to the HPA signaling axis [50,51], and is consistently reported to be increased in socially isolated rodents [69]. In human studies, increased serum cortisol suggestive of HPA axis hyperfunction has been found in individuals with schizophrenia [70]. Importantly, a past study investigating the effect of social isolation on a transgenic mouse model of psychiatric disorder (DISC1-DN-Tg-PrP) found significant increases in plasma corticosterone as a result of G x E interaction [24]. In this study, differential regulation of DNA methylation through glucocorticoid receptor signaling was found in subsets of ventral tegmental area (VTA) dopaminergic neurons projecting to different areas (mesolimbic, mesocortical), revealing specific subsets of VTA neurons that are targeted by corticosterone signaling. Later studies have found that synaptic functions in the PFC network can be influenced by glucocorticoids ether directly through glucocorticoid signaling or indirectly through the modulation of upstream projecting neurons [71]. Increased levels of corticosterone in *Kpna1* KO mice could affect similar regions such as the PFC or hippocampus, causing epigenetic changes and disruption of downstream neural pathways.

Recent studies exploring diagnostic biomarkers in human patients have highlighted the role of systemic signal molecules such as cytokines, hormones, and their receptors in psychiatric disorders [72–74]. Alterations in neuroendocrine pathways such as HPA axis signaling are known to influence circulatory cytokine levels [29,53]. In this study, we assessed the impact of genetic and environmental factors on the circulatory levels of such stress-associated systemic signal molecules (cytokines, hormones, and receptors) through a multiplex immunoassay, finding several species of signal molecules that were altered in *Kpna1* KO and/or isolation stress mice.

Of the 11 species detected in the multiplex immunoassay, both group-housed and isolated *Kpna1* KO mice exhibited higher plasma LIX levels than WT controls. LIX (CXCL5) is a CXCR2 ligand chemokine (cytokine responsible for chemotactic recruitment of leucocytes into tissue) involved in neuroinflammation, BBB collapse, and neutrophil infiltration into the brain [75]. Furthermore, LIX has been associated with schizophrenia in a large-scale assessment of plasma protein biomarkers in patients [73]. Recently, neuro-immune interactions involved in the regulation of various behaviors has become an area of increasing interest [76,77], and investigations on other rodent social stress paradigms such as repeated social defeat have revealed neuro-immunological alterations such as increased neutrophil mobilization [78] and increased brain monocyte infiltration [79], as potential factors causing abnormal behaviors in these models. As increases in plasma LIX levels can cause increases in chemokine signaling and leucocyte chemotaxis/infiltration, a potential mechanism of increased LIX levels in *Kpna1* KO affecting behavior may be an increased level of leucocyte infiltration into the brain after social stress. As our measurements are only from LIX in circulation, further evidence on LIX production/secretion in the brain and other organs in *Kpna1* KO mice is necessary to determine the connections between *Kpna1* KO and LIX alteration.

In our measurements of plasma prolactin, a G x E interaction was found on plasma prolactin levels, where WT mice had higher prolactin levels compared to other groups. prolactin is a protein hormone secreted from the anterior pituitary involved in the regulation of physiological functions including reproduction, metabolism, and maternal behavior [80,81], with more recent evidence suggesting additional regulatory roles such as HPA axis regulation [82,83] and suppression of anxious/depressive behavior [84] in rodents. Administration of social isolation stress has been found to disrupt circadian fluctuations of prolactin in rats, causing circulatory prolactin levels to be low throughout the day compared to unstressed controls [85,86]. Although the effects of *Kpna1* deletion on prolactin secretion, along with the effects of circulatory prolactin on the behaviors caused by G x E interaction in this study (FS, IA) have not been investigated yet, the decreased prolactin levels found in our study may underlie changes in signaling pathways and feedback loops involved in stress response and behavioral regulation. Further insight into the regulatory roles of prolactin on HPA axis regulation and behavior may uncover converging points for the adverse effects of genetic and environmental stressors.

As prolactin secretion from the pituitary is negatively regulated by dopamine signaling from the hypothalamus [87], the main focus on prolactin in clinical settings with regard to psychiatric disorders has been antipsychotic induced hyperprolactinemia, an adverse effect caused by the administration of common antipsychotics which target dopamine D2 receptors [88]. However, emerging evidence has suggested additional connections between prolactin and psychiatric disorders, where negative correlations have been found between systemic prolactin levels and measures of positive symptoms in schizophrenia patients [89]. Future studies may uncover further connections between systemic prolactin signaling and behavioral regulation.

Exposure to adolescent social isolation stress in rodents has been proposed as a paradigm to mimic social stress factors [28] known to affect human patients with psychiatric disorders such as schizophrenia and pathological social withdrawal (*Hikikomori*) [90,91], and changes in anxiety-like behavior, depression-like behavior, recognition memory, and PPI following social isolation stress have previously been reported across numerous studies [92]. Our study provides further support for the findings of many of these studies, demonstrating exposure to social isolation stress alone to result in increased anxiety-like behavior in the OFT, impaired aversive learning and/or memory in IA, reduced sensorimotor gating in PPI, and increased depression-like behavior in FS. In addition to these behavioral changes, past studies have reported social isolation in rodents to result in changes in corticosterone [52]. In our study we measured the levels of several stress-associated signal molecules and found for the first time that mice that experienced adolescent social isolation showed altered levels of CCL11, TNFRII, and TIMP-1. CCL11 was increased, whereas TNFRII and TIMP-1 were decreased, in socially isolated mice. CCL11 (Eotaxin-1) is a chemokine known to enhance excitotoxicity [93] and is associated with neurological aging in mice [94] and psychiatric disorders in humans [95]. TNFRII acts as a receptor for TNF and is involved in the neuroprotective roles of TNF signaling [96]. Changes in TNF signaling in the brains of patients with psychiatric disorders (schizophrenia, bipolar disorder) have been an area of therapeutic interest [97]. TIMP-1 is a matrix metalloprotease inhibitor involved in the regulation of neuroinflammation through matrix metalloprotease regulation [98]. Future research is required to ascertain the mechanisms underlying stress-associated changes in these signal molecules and how they may act to regulate behavior.

Past reports have characterized the widespread expression of *Kpna1* across the brain [2] and its regulatory roles in neuronal differentiation [7,8]. Our study expands on these previous reports by providing novel evidence that genetic alterations in *Kpna1* can combine with environmental stress factors to cause behavioral abnormalities associated with several psychiatric disorders. These findings appear to support previous evidence from genetic studies of human psychiatric disorder patients revealing a *de novo* nonsense mutation of *KPNA1* to be associated with schizophrenia [16]. However, it is important to note that similar *de novo* mutations have not been identified by further screening of schizophrenia patients [99], and *KPNA1* is yet to be identified in large-scale GWAS studies of human schizophrenia patients despite being proposed as an important regulator of neuronal differentiation and behavior in mice. Future attempts to include the effects of environmental stress factors in large-scale analysis may uncover the effects of previously unknown G x E interactions and highlight the contributions from less common genetic risk factors.

Further assessment of the molecular mechanisms underlying the behavioral abnormalities seen in *Kpna1* KO mice may be an effective approach for elucidating the complex molecular mechanisms underlying human pathology, along with advancing the understanding of Importin αs in physiological contexts. Finally, the finding that social isolation stress in *Kpna1* KO mice resulted in an augmentation of some behavioral alterations demonstrates the effectiveness of this mouse models to dissect the interactive effects of individual genetic and environmental factors in relation to psychiatric disorders.

## Supporting information

S1 Table ANOVA results. F and P values for ANOVAs.
(XLSX)

S2 Table Number of mice and number of outliers. The number of mice used for each experiment and number of outliers excluded.
(XLSX)
